# Post-surgical Thyroid Bed Pyoderma Gangrenosum Mimicking Recurrent Papillary Thyroid Carcinoma

**DOI:** 10.3389/fendo.2019.00253

**Published:** 2019-04-18

**Authors:** Alessia Dolci, Rita Indirli, Giovanni Genovese, Federica Derlino, Maura Arosio, Angelo Valerio Marzano

**Affiliations:** ^1^Endocrinology Unit, Fondazione IRCCS Cà Granda Ospedale Maggiore Policlinico, Milan, Italy; ^2^Department of Clinical Sciences and Community Health, University of Milan, Milan, Italy; ^3^Dermatology Unit, Fondazione IRCCS Ca' Granda Ospedale Maggiore Policlinico, Milan, Italy; ^4^Department of Pathophysiology and Transplantation, University of Milan, Milan, Italy; ^5^Dermatology Unit, Department of Health Sciences, ASST Santi Paolo e Carlo, University of Milan, Milan, Italy

**Keywords:** pyoderma gangrenosum, differentiated thyroid carcinoma, papillary thyroid carcinoma, pathergy, thyroidectomy

## Abstract

**Background:** Pyoderma gangrenosum (PG) is a rare inflammatory disease presenting with chronic-recurrent cutaneous ulcers histopathologically hallmarked by neutrophilic infiltrates, which may occur more frequently at sites of surgical traumas. The disease is habitually limited to the skin, but it can virtually involve any organ. Nevertheless, no prior cases of PG involving the thyroid bed have ever been reported.

**Case Report:** A bilateral PG of the breast was diagnosed in a 51-year-old woman and treated with intravenous methylprednisolone pulse-therapy and cyclosporine, with partial improvement. During the hospitalization, cytological examination of two hypoechoic thyroid nodules by fine-needle aspiration (FNA) was consistent with thyroid carcinoma. After total thyroidectomy, histopathology confirmed a papillary thyroid cancer (PTC), and radioactive iodine ablation was performed. At 12-month ultrasonographic follow-up, two hypoechoic avascular areas localized in the empty thyroid bed raised the suspect of PTC recurrence. However, (i) undetectable levels of thyroglobulin without anti-thyroglobulin antibodies, (ii) neutrophilia and increased inflammatory marker levels, and (iii) cytological examination of FNA showing numerous neutrophils induced to suspect thyroid bed PG infiltration. An *ex juvantibus* approach with high-dose methylprednisolone led to dimensional reduction of the hypoechoic areas on ultrasonography, thus confirming the hypothesis of thyroid bed PG.

**Conclusion:** This case of thyroid bed PG supports the idea that PG reflects a cutaneous phenotype encompassed in the spectrum of systemic neutrophilic diseases. Endocrinologists should be aware that thyroid bed PG involvement is an albeit rare differential diagnosis to consider in patients who had undergone thyroid surgery, especially with a history of PG.

## Introduction

Papillary thyroid carcinoma (PTC), the main variant of differentiated thyroid carcinoma (DTC), is the most common thyroid cancer, accounting for 70–80% of all thyroid carcinomas and occurring predominantly in women, generally with a good prognosis (1). PTC is characterized by a steady and continuous increase in the incidence rate, which has more than doubled within the past three decades all over the world (2).

Pyoderma gangrenosum (PG) is a rare chronic-relapsing inflammatory disease clinically characterized by single or multiple skin ulcers with undermined erythematous-violaceous borders usually developing on the lower extremities (3). It is nowadays regarded as autoinflammatory in origin and classified in the spectrum of the so-called neutrophilic dermatoses, which are a group of conditions hallmarked by accumulation of mature neutrophils in the skin (4–6). Albeit rarely, in this disease almost any organ system can be involved by sterile neutrophilic infiltrates, with the lungs being the most commonly affected site (3, 4). This condition may be idiopathic or associated with underlying disorders, notably inflammatory bowel diseases, rheumatological forms or neoplasms, particularly of lymphoreticular origin (3, 4). PG is typically accompanied by a skin hyperreactivity leading to the pathergy phenomenon, which is the occurrence of disease manifestations at sites of traumas, either accidental or surgical ones (3, 7).

Herein, we report an extraordinary case of a female patient with PG manifesting as classic ulcerative skin lesions associated with a mass in an empty thyroid bed after total thyroidectomy for PTC. This extracutaneous infiltrate mimicked a recurrence of PTC on ultrasound (US) examination, making challenging the diagnosis and management of this unique case.

## Materials and Methods

Serum thyroglobulin (Tg) levels were measured by electrochemiluminescence sandwich immunoassay (ECLIA, Elecsys Tg II, Roche®, Mannheim, Germany) with an analytic sensitivity of 0.04 ng/ml. Serum anti-Tg antibodies levels were measured by fluorescence enzyme immunoassay (EliA; Thermo Fisher Scientific, Life Technologies Italia®, Monza, Italy; normal <40 IU/ml). Thyroid ultrasound was performed at each time of follow-up using a 14–16-MHz linear probe by the same endocrinologist.

Skin biopsy including both the edge and bed of the ulcer was performed. The skin specimen was fixed in 10% buffered formalin, embedded in paraffin, and sectioned into 3-μm sections that were stained with haematoxylin-eosin.

Written informed consent was obtained from the patient for the publication of this case report and any potentially-identifying images and/or information.

## Case Description

**Figure 1:**
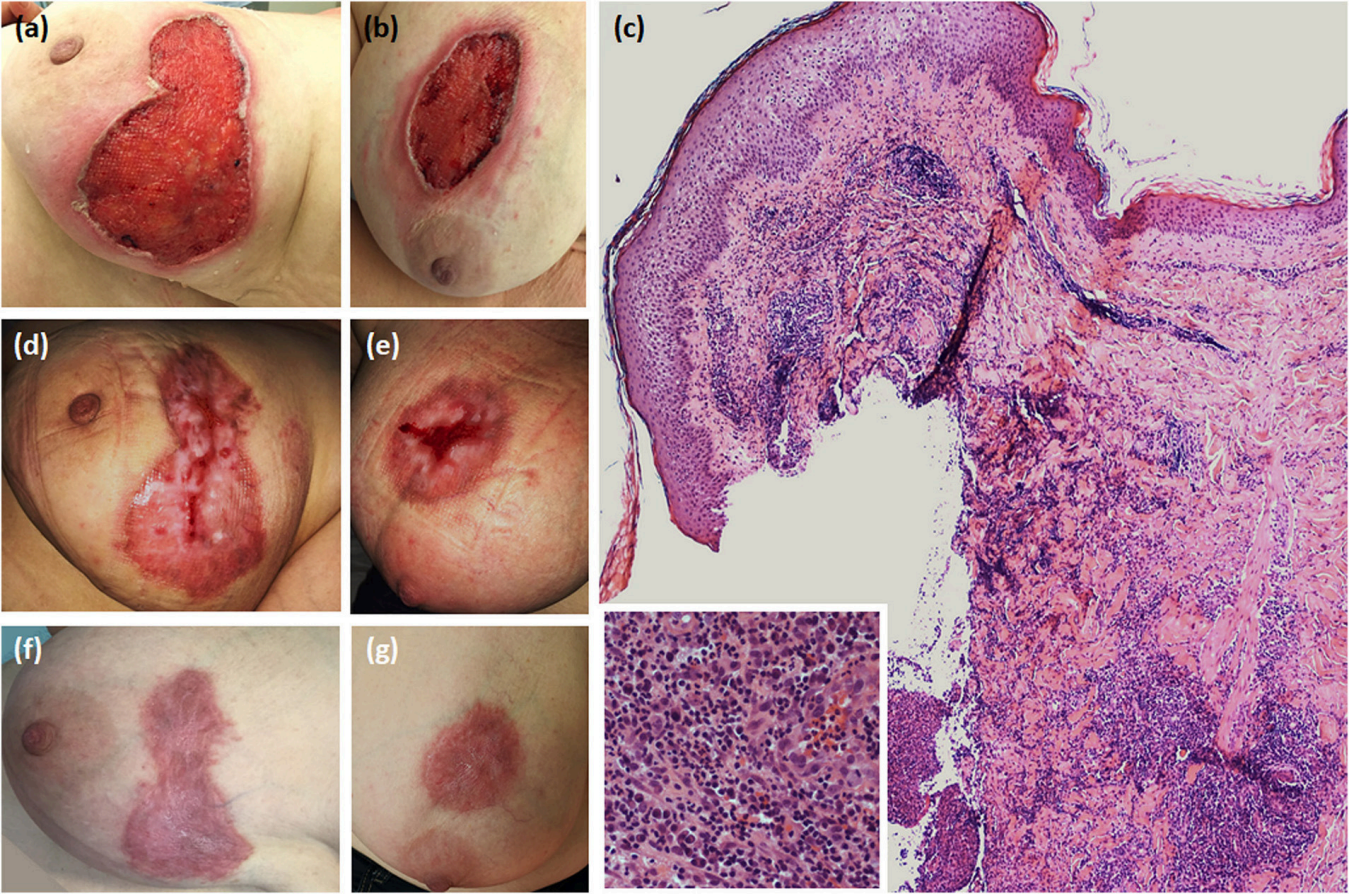
Dermatological and histopathological characteristics of pyoderma gangrenosum. **(a,b)** Ulcerative lesions showing necrotic base and erythematous-violaceous undermined borders on the right and left breast, respectively; **(c)** Skin histology revealing epidermal necrosis and a dermal-hypodermal inflammatory infiltrate mainly consisting of neutrophils (haematoxylin-eosin, original magnification × 100) (in the box, a magnified detail of the inflammatory infiltrate); **(d,e)** Partial healing after therapy with pulsed intravenous methylprednisolone, followed by the combination of prednisone and cyclosporine given orally; **(f,g)** Complete healing with hypertrophic aspects.

A 51-year-old woman was hospitalized for the occurrence of two bilateral painful skin ulcers with undermined, erythematous-violaceous edges in the mammary region, which had begun 6 months before and had rapidly developed from pustular lesions (Figures 1a,b). The skin lesions were refractory to antiseptic and antibiotic therapies which had been administered in another dermatology department.

Skin biopsy showed a dermal-hypodermal neutrophilic infiltrate, suggesting PG (Figure 1c). Laboratory work-up ruled out any underlying inflammatory condition. Pulse-therapy with intravenous methylprednisolone 125 mg daily for 5 consecutive days was given with clinical improvement, followed and followed by prednisone at progressively tapering dosages in combination with cyclosporine 300 mg daily, inducing progressive healing of the lesions (Figures 1d–g). During hospitalization, chest computerized tomography revealed a multinodular goiter. Neck US disclosed a 12.4 mm solid hypoechoic nodule in the upper pole of the left thyroid lobe, and another 8.5 mm hypoechoic nodule in the lower pole of the same lobe. Fine-needle aspiration (FNA) of the dominant nodule was performed, and the cytological exam resulted in Tir4 category according to 2014 SIAPEC (Società Italiana di Anatomia Patologica) classification, equivalent to “suspicious for thyroid carcinoma” (8). A total thyroidectomy was performed, and the histological examination was consistent with multicentric classical PTC, with focal extension to extra-thyroidal soft tissues and surgical resection margins. Histopathologic stadium was pT3(m)Nx according to AJCC (American Joint Committee on Cancer) TNM VII Edition Staging System (9).

Postoperatively, she received radioactive iodine ablation treatment with 3700 MBq under human recombinant α-thyrotropin stimulation (Thyrogen®). The post-treatment Whole Body Scan showed no uptake outside the thyroid bed. The stimulated Tg serum value was 2.6 ng/ml.

Given these findings, the patient was classified at intermediate risk of recurrence according to 2015 ATA score system. Due to the neoplasm, cyclosporine was replaced by immunomodulating therapy with dapsone, without recurrence of skin manifestations.

**Figure 2:**
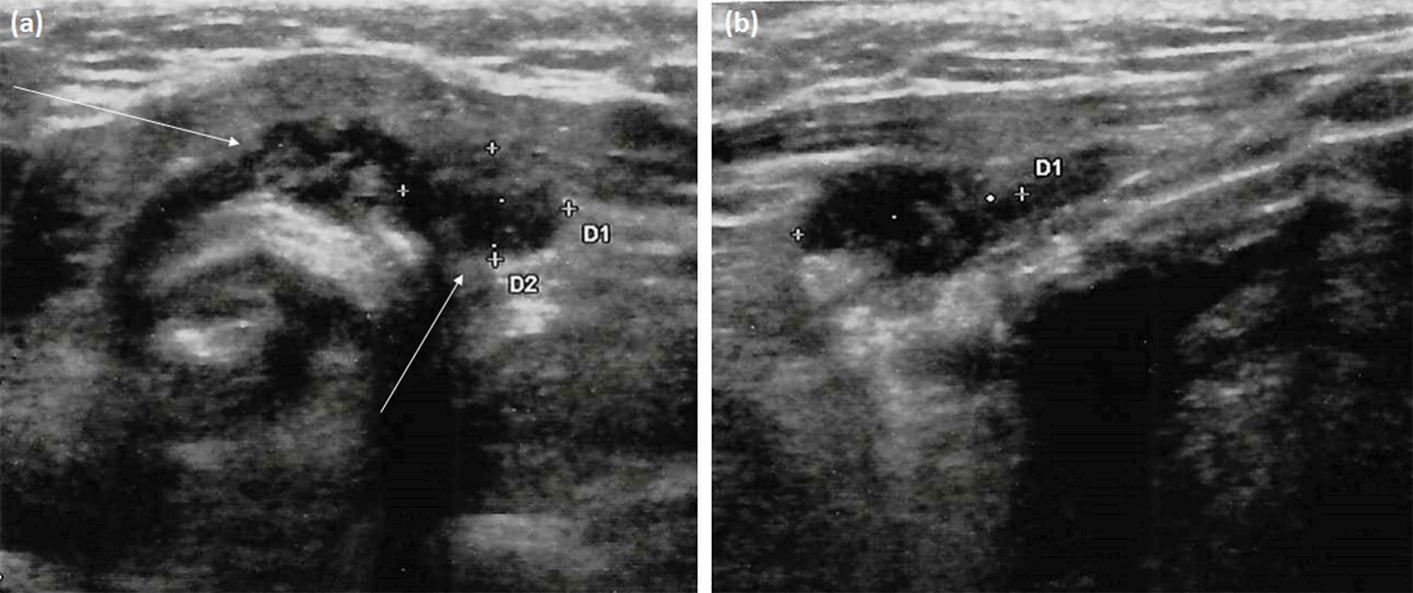
Neck ultrasonography performed at 12-month follow-up visit after thyroidectomy. **(a)** Transversal scan shows two adjacent left paratracheal lesions (arrows). These marked hypoechoic areas have ill-defined margins but not microcalcifications; **(b)** Longitudinal scan depicts the elongated shape of the paratracheal lesion (lateral one) and its parallel orientation to the dermis without deformation of surrounding tissues, unlike true focal masses.

As shown in Table 1, at the 6-month follow-up examination, there was no clinical, biochemical or US evidence of tumor persistence or recurrence. Serum Tg concentrations were undetectable during replacement thyroxine therapy (unstimulated Tg measured several times by an high-sensitive assay always <0.04 ng/mL) in the absence of interfering anti-Tg antibodies. However, US examination of the neck performed 12 months after thyroid surgery revealed two hard hypoechoic avascular areas with irregular margins in the thyroid bed, which measured 16 mm and 15 mm in their maximum diameter, respectively (Figure 2). No suspicious lymph nodes were detected. Serum Tg levels were still undetectable, with negative anti-Tg antibodies. FNA of the two above-mentioned lesions was performed under the impression of PTC recurrence; the cytologic specimen revealed numerous neutrophils, together with scattered erythrocytes, lymphocytes, histiocytes, and few thyrocytes without cytological alterations. Gram staining as well as bacterial and mycobacterial cultures of the FNA specimen were negative. Blood exams revealed increased inflammatory markers and neutrophilia.

**Table 1:** Laboratory tests and neck ultrasound scans performed during the follow-up.

**Time-point**	**TSH (mIU/L)**	**Tg (ng/mL)**	**Anti-Tg antibodies (IU/mL)**	**ESR (mm)**	**CRP (mg/dL)**	**Absolute neutrophil count (× 10^**9**^/L)**	**Neck ultrasound scan**
6 months after radioiodine ablation	2.1	<0.04	<12	N/A	N/A	N/A	No suspicious lesions in the thyroid bed, no suspicious lymph nodes
12 months after radioiodine ablation	0.89	<0.04	<12	60	1.17	3.22	Two hypoechoic avascular areas with irregular margins in the thyroid bed, 16 mm and 15 mm in their maximum diameter, respectively; no suspicious lymph nodes
One week after pulse intravenous methylprednisolone	0.085	<0.04	<12	28	0.6	7.02	The two hypoechoic avascular areas in the thyroid bed reduced to 10.9 mm and 15 mm in their maximum diameter, respectively; no suspicious lymph nodes
One month after pulse intravenous methylprednisolone	N/A	N/A	N/A	Normal	Normal	Normal	The two hypoechoic areas furtherly reduced to 8.6 mm and 9.2 mm in their maximum diameter, respectively; no suspicious lymph nodes
Ten months after pulse intravenous methylprednisolone^a^	1.59	<0.04	<12	N/A	N/A	N/A	The two hypoechoic areas are no more visible in the thyroid bed; no suspicious lymph nodes

TSH, Thyroid Stimulating Hormone; Tg, thyroglobulin, analytic sensitivity 0.04 ng/ml; Anti-Tg antibodies, anti-thyroglobulin antibodies, analytic sensitivity 12 IU/mL; ESR, Erythrocyte Sedimentation Rate, normal values <20 mm; CRP, C-Reactive Protein, normal values <0.5 mg/dl; N/A, not available.

a *During current therapy with oral prednisone and cyclosporine*.

**Figure 3:**
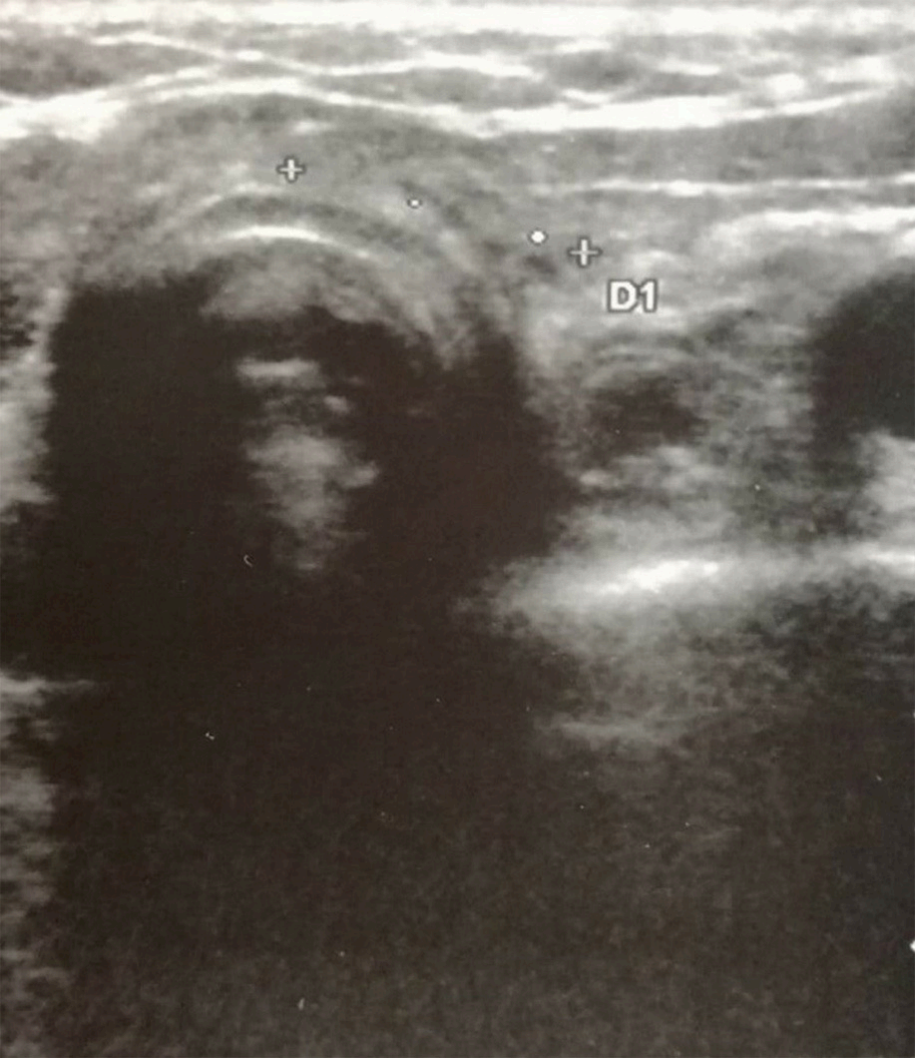
Neck ultrasonography performed at 10-month follow-up visit after the second cycle of intravenous methylprednisolone treatment shows complete regression of the two hypoechoic areas in the thyroid bed.

The clinical, biochemical and cytological clues were consistent with PG infiltration of the thyroid bed. Therefore, the patient underwent again pulse therapy with intravenous methylprednisolone under a strict US follow-up. Following steroid treatment, the two cervical lesions appeared to be dramatically reduced on neck US (with one lesion passing from 10.9 mm in maximum diameter after the first week, to 8.6 mm after 1 month; the second cervical area passed from 15 to 9.2 mm in maximum diameter at the same time points). Both lesions mentioned above were no more visible after 10 months (Figure 3) confirming thyroid bed involvement by PG.

## Discussion

Detecting a mass in the thyroid bed of a patient who had a past medical history of PG and thyroid cancer raises both the possibility of relapse of PG or local recurrence of PTC. PG of the thyroid bed has not been previously reported, and on ultrasound imaging the morphology of PG lesions appeared very similar to thyroid tissue. Essential clues for differentiating deep tissue localization of PG and a recurrence of PTC on US is the parallel orientation of the former, the absence of bright echogenic spots, the elongated and poorly defined borders and its proximity to a skin PG wound (7, 10).

To explain the exceptional association of these two different diseases, different pathogenetic hypotheses may be postulated. Firstly, PG can be a paraneoplastic manifestation of an underlying tumor, particularly haematolymphoid malignancies (11). However, DTC is rarely related to paraneoplastic syndromes (12, 13), and in the present case PG relapsed upon reaching complete remission of PTC, thus making the relation between PG and PTC unlike in our patient.

Common genetic basis may alternatively be involved. Germline mutations in different genes involved in the innate immune response have been identified in autoinflammatory diseases (4). Similarly, a number of somatic mutations have been found to occur in DTC (14). However, the cellular pathways involved in the two conditions look different, and it is difficult to support a common monogenic basis. Nevertheless, dedicated studies in this field are lacking, and we cannot definitely exclude that a polygenic background may eventually predispose to both conditions.

Finally, a conceivable hypothesis to explain the occurrence of PG at the thyroid bed following surgery for PTC is represented by the *pathergy* phenomenon. Pathergy consists in the development of new skin lesions or the aggravation of existing ones after trauma (15). Pathergy has a pivotal role in inducing new post-surgical *skin* PG in patients with a preexisting diagnosis of PG. However, while *cutaneous* PG has been frequently reported after surgical procedures involving the skin, especially breast surgery (16), visceral PG involvement is usually not related to previous surgery. This may be due to the fact that visceral PG is a very rare condition and its diagnosis may be difficult to reach. On the other hand, cases of visceral PG secondarily involving the skin have been reported, as in a patient with lung PG mimicking a lung carcinoma who, 3 months after a sleeve lobectomy and chest-wall resection, developed skin PG ulcers at the site of the surgical wound (17).

Therefore, we suppose that pathergy phenomenon may have contributed to the development of post-surgical visceral PG, even though its exact pathogenesis is still elusive. This case report reinforces the concept that PG may be regarded as a neutrophilic systemic disease, which may be associated with infiltration of sterile neutrophilic infiltration of internal organs (4). The rare extracutaneous neutrophilic involvement in PG (18), has been mainly described as culture-negative pulmonary lesions, but also bone, joint, central nervous system, spleen, kidney, and digestive tract involvements have been reported (18).

A role for radioiodine therapy in this context is not defined, as no specific association between PG and radioiodine has been reported so far.

There have been many reports of non-thyroidal lesions which can be mistaken for thyroid lesions on US (10). However, although it has never been reported that a PG asymptomatic localization might mimic a recurrent thyroidal lesion, establishing the correct diagnosis is crucial, since PG could be exacerbated by an unnecessary surgical intervention potentially leading to pathergy. On the other hand, surgery or radioactive iodine ablation would have been the first-choice treatments if a thyroid cancer recurrence had been ascertained.

In conclusion, a multidisciplinary approach is needed to correctly diagnose and manage these rare cases of extracutaneous PG.

## Ethics Statement

This study was exempt from ethical approval procedures being a case report that describes the clinical course and outcome of a single patient who was referred to our outpatient clinic.

## Patient Consent

The patient provided written consent to have her case published for the purpose to improve the medical knowledge.

## Author Contributions

AD, MA, and AM performed patient follow-up, clinical diagnosis, and manuscript preparation. RI, AD, and GG performed data interpretation and manuscript preparation. FD collected the patient's clinical information and contributed to manuscript preparation. AM and MA performed the critical revision of the manuscript. All authors approved the manuscript and this submission.

### Conflict of Interest Statement

The authors declare that the research was conducted in the absence of any commercial or financial relationships that could be construed as a potential conflict of interest.
